# Interspecies Interactions Reverse the Hazard of Antibiotics Exposure: A Plankton Community Study on Responses to Ciprofloxacin hydrochloride

**DOI:** 10.1038/s41598-017-02593-4

**Published:** 2017-05-24

**Authors:** Changyou Wang, Ziyang Wang, Yong Zhang, Rongguo Su

**Affiliations:** 1 School of Marine Sciences, Nanjing University of Information Science and Technology, Nanjing, 210044 China; 2 Qingdao No. 19 Middle School of Shandong Province, Qingdao, 266021 China; 3 Institute of Marine Science and Technology, Shandong University, Ji-nan, 250100 China; 4 College of Chemistry and Chemical Engineering, Ocean University of China, Qingdao, 266100 China; 5 Jiangsu Research Center for Ocean Survey Technology, Nanjing University of Information Science and Technology, Nanjing, 210044 China

## Abstract

The ecotoxicological effects of Ciprofloxacin hydrochloride (CIP) were tested on population densities of plankton assemblages consisting of two algae (*Isochrysis galbana* and *Platymonas subcordiformis*) and a rotifer (*Brachionus plicatilis*). The *I. galbana* showed a significant decrease in densities when concentrations of CIP were above 2.0 mg L^−1^ in single-species tests, while *P. subcordiformis* and *B. plicatilis* were stable in densities when CIP were less than10.0 mg L^−1^. The equilibrium densities of *I. galbana* in community test increased with CIP concentrations after falling to a trough at 5.0 mg L^−1^, showed a completely different pattern of *P. subcordiformis* which decreased with CIP concentrations after reaching a peak at 30.0 mg L^−1^. The observed beneficial effect was a result of interspecies interactions of trophic cascade that buffered for more severe direct effects of toxicants. The community test-based NOEC of CIP (2.0 mg L^−1^), embodying the indirect effects, was different from the extrapolated one derived by single-species tests (0.5 mg L^−1^), but all lacked confidence interval. A CIP threshold concentration of obvious relevance to ecological interaction was calculated with a simplified plankton ecological model, achieving a value of 1.26 mg L^−1^ with a 95% bootstrapping confidence interval from 1.18 to 1.31 mg L^−1^.

## Introduction

Antibiotics were used in both human and veterinary medicine since 1940s and the annual amount have been up to 200,000 tons in recent years^1^. A part of the total amount were released to the environment by a number of routes, such as manufacturing process, aquaculture treatments, faeces excreted to the sewer system, direct dumping of these drugs in the sewage both in residences and in human and veterinary hospitals^2–4^. The widespread use of antibiotics has led to a concern in their post-therapeutic effects and broad-scale environmental monitoring studies. It has been well recognized that antibiotics in the environment could select more adapted genes to the new environmental characteristic or may even induce mutations of bacterial pathogens^3, 5, 6^. Another growing concern about antibiotics in the environment is focused on their ecological effects. Many papers have documented the toxic effects of antibiotics on aquatic and soil species and discussed the factors driving the effect of antibiotics contaminations on species assemblages^3, 7^. However, previous studies are mainly revolved in single-species toxicity tests. Few studies have taken into account the potentially indirect effects of antibiotics on structure of ecosystems. Studies have proved the indirect effects of ecological reactions on the toxic effects are significant^8, 9^. Though the present antibiotics concentration in environment were not believed to pose a big direct risk for toxicity for species, the indirect effect induced by antibiotics may alter species abundances, change community composition, and weaken or enhance direct toxic effects of antibiotics^3^.

Among all kinds of antibiotics, fluoroquinolones is a representative of synthetically produced antibiotics and one of the most important classes of antibiotics, as a result of their activities against a broad spectrum of bacteria and their annual global sales^10, 11^. The environmental monitoring studies have investigated them at concentrations ranging from mg L^−1^ to ng L^−1^ ^12, 13^. Ciprofloxacin hydrochloride (CIP) is one of common fluoroquinolones. Though CIP is a primary degradation product of enrofloxacin, it is not further readily biodegradable in water. Moreover, CIP is readily transported into the environment via discharge of wastewater and direct runoff and used worldwide in aquaculture applications^1, 13^. However, until recently very limited data have been available on the ecological effects of CIP on marine planktons in the ocean^14^.

One of the targets for the protection of ecosystem is to prevent the obvious variation in every species biomass. The maximum concentration which does not affect the biomass amount of the test species is often necessary to be determined. The No Observed Effect Concentration (NOEC) is usually a statistic used as an estimate of this concentration. Although NOEC is easy to determine and easy to understand, it is only able to take the value of tested concentrations and is impossible statistically to construct confidence interval for it at present. The representative shortcomings have been shown in a series of papers^15–17^. The ecotoxicologists have advocated replacing gradually the NOEC and using a regression-based estimation procedure as a replacement proposal^14, 15, 18, 19^.

In this paper, a community consisting of *Isochrysis galbana*, *Platymonas subcordiformis* and *Brachionus plicatilis*, is used in an attempt to evaluate toxic effects for CIP exposure and to discuss the impact of ecological reactions on toxic effects of CIP. The threshold concentration of CIP for the customized community is calculated with a simplified plankton ecological model and is compared with the NOEC determined with the same data in this study.

## Materials and Methods

### Chemicals

The CIP used in this study was purchased from Beta Pharma Ltd., Shanghai, China and dissolved by ultrapure water directly, with the stock solution concentration at 5 g L^−1^. Ultraviolet Spectrophotometry (UV-2102PCS, Unico (Shanghai) Instrument Co., Ltd) was used to determine CIP concentrations at 275 nm. The analytical limits of detection were 1.5 μg mL^−1^ and the relative standard deviations (RSD) values ranging from 1.6 to 2.5%^20^. The test concentrations of the CIP was 0, 2, 5, 10, 30, 50 and 70 μg mL^−1^, which were determined with a preliminary experiment, and the corresponding measured concentrations ranged from 95% to 99% of the nominal concentration at the beginning of tests and from 93% to 88% in the end of tests. Nitrate, phosphate, vitamins and trace elements use in f/2 medium were bought from Shanghai Chemical Reagents Co., China.

### Eco-toxicological effect test

The toxic effects of CIP were studied by single-species toxicity tests and community toxicity test. In algal single-species toxicity tests (n = 3), *I. galbana* and *P. subcordiformis* were cultivated in 300 mL conical flask, respectively, and placed in an incubator with a constant temperature of 20 °C, a photoperiod of 12 h light:12 h dark and a photon irradiance of ca. 60 μmol m^−2^ s^−1^. The 150 ml f/2 culture medium, prepared from NaNO_3_, Na_2_HPO_4_, vitamins and trace elements in autoclaved natural seawater, was poured in the culture flask. The conical flasks were shaken two times a day during the tests, one in the morning and one in the evening. The seawater with a salinity of 25 psu was sourced from the Yellow Sea near Rudong city. The initial incubation density was determined to be 0.027 × 10^6^ cells mL^−1^ for *P. subcordiformis* and 0.2 × 10^6^ cells mL^−1^ for *I. galbana* in order to get the same biomasses and keep basically matched competition at the beginning of tests^21^. The prepared solution of CIP was added to culture flasks within an hour after algae inoculation. The toxicity tests for algae ended when their densities began to fall, and the longest time was 14 days. During the test period, 3-mL water samples were taken from the conical flask every day and fixed by the addition of Lugol’s solution. Shake up culture medium before samples were taken. A light microscopy (XLE-2, 3DFAMILY Technology Co., Ltd., Nanjing, China) was used to determine Algal species and cell number. The same culture conditions and CIP concentrations were used in the algae bi-species toxicity tests.

In rotifer single-species toxicity tests (n = 3), the *B. plicatilis* were also cultured in 300 mL conical flask and fed every 24 h with the algae *Chlorella sp*. at a density of 1 × 10^6^ cells mL^−1^ during the test^22^. Culture flasks were put in an incubator with the same temperature and photoperiod as the algae single-species toxicity tests. The *B. plicatilis* were incubated from an initial density of 10 individuals mL^−1^ in 150 ml culture medium. 3-mL water samples were taken from culture flasks every day during the test period and densities of the survival rotifers were measured by the microscopy with a 1-mL plankton counting chamber. The tests lasted until the rotifer densities began to fall, the longest one was 21 days in this study.

In the customized plankton community toxicity test (n = 3), the initial density was 5 individuals mL^−1^ for *B. plicatilis*, 0.027 × 10^6^ cells mL^−1^ for *P. subcordiformis* and 0.2 × 10^6^ cells mL^−1^ for *I. galbana*. The motile individuals in the same culturing conditions were selected as initial rotifers. The community were cultured in 1 L conical flask of 0.5 L culture medium and put in an incubator with the same conditions as the single-species toxicity tests. During the test, 3-mL water samples were taken from culture flasks every 3 days in the first 21 days and every 5 days afterwards. The tests lasted 31 days. The samples were dropped in plankton counting chamber and observed with microscopy to determine their species and densities.

### The method used to calculate the benchmark of CIP for the plankton community

The ecotoxicological effect of CIP in algae single-species toxicity tests was estimated with the endpoint of carrying capacity, which was calculated with logistic growth model^17^. The ecotoxicological effect in rotifer single-species toxicity test was estimated with the endpoint of survival rate^23^. The ecotoxicological effect in the customized plankton community toxicity test was estimated with the endpoint of equilibrium biomasses of three species, which was supposed to be approached if the densities of three species were not changed significantly in 5 days^8^. The averages of biomass for each species in 5 days were calculated to be equilibrium biomasses.

The population-NOEC of CIP for each plankton population and a community-NOEC for the customized plankton community are determined by a one-sided t-test (α = 5%), respectively. In this study, the community-NOEC was obtained by comparing equilibrium biomasses of three species in treatments with that in the control.

A simplified plankton ecological model was used to calculate the threshold concentration for the customized plankton community. The plankton ecological model was constructed by integrating the Logistic growth equation and Lotka-Voterra equation into a competition-grazing model^17, 24^. In addition, the algal density restrictions were accounted for in this model. The simplified plankton ecological model equations are provided below:

1 $$\frac{d{P}_{1}}{dt}={P}_{1}({r}_{1}-{a}_{11}{P}_{1}-{a}_{12}{P}_{2}-{a}_{13}Z)$$

2 $$\frac{d{P}_{2}}{dt}={P}_{2}({r}_{2}-{a}_{21}{P}_{1}-{a}_{22}{P}_{2}-{a}_{23}Z)$$

3 $$\frac{dZ}{dt}=Z(\,-\,{r}_{3}+{a}_{31}{P}_{1}+{a}_{32}{P}_{2})$$

According to the non-linear dynamic results of the ecosystem, the simplified plankton ecological model only yielded a positive asymptotic equilibrium point E* (P_1_*, P_2_*, Z*) when the plankton biomasses and model coefficient matrix were consistently greater than zero, indicating that the customized plankton ecosystem would continuously survive.

4 $${P}_{1}^{\ast }=\frac{{a}_{12}{a}_{23}{r}_{3}+{a}_{13}{r}_{2}{a}_{32}-{r}_{1}{a}_{23}{a}_{32}-{a}_{13}{a}_{22}{r}_{3}}{{a}_{12}{a}_{23}{a}_{31}+{a}_{13}{a}_{21}{a}_{32}-{a}_{11}{a}_{23}{a}_{32}-{a}_{13}{a}_{22}{a}_{31}}$$

5 $${P}_{2}^{\ast }=\frac{{r}_{1}{a}_{23}{a}_{31}+{a}_{13}{a}_{21}{r}_{3}-{a}_{11}{a}_{23}{r}_{3}-{a}_{13}{r}_{2}{a}_{31}}{{a}_{12}{a}_{23}{a}_{31}+{a}_{13}{a}_{21}{a}_{32}-{a}_{11}{a}_{23}{a}_{32}-{a}_{13}{a}_{22}{a}_{31}}$$

6 $${Z}^{\ast }=\frac{{a}_{11}{a}_{22}{r}_{3}+{a}_{12}{r}_{2}{a}_{31}+{r}_{1}{a}_{21}{a}_{32}-{a}_{11}{r}_{2}{a}_{32}-{a}_{12}{a}_{21}{r}_{3}-{r}_{1}{a}_{22}{a}_{31}}{{a}_{12}{a}_{23}{a}_{31}+{a}_{13}{a}_{21}{a}_{32}-{a}_{11}{a}_{23}{a}_{32}-{a}_{13}{a}_{22}{a}_{31}}$$

The biomasses in equilibrium for every population were calculated with the parameters used in the simplified plankton ecological model, which were provided in the supporting document (appendix). The calculated biomasses in equilibrium diverged from the original equilibrium point as the CIP concentration increased. In this study, the maximum CIP concentration at which the biomasses in equilibrium did not diverge from the original, that is, was not of significant difference, was defined as the threshold concentration for equilibrium point (TCEP). The significance measurement between the calculated biomasses in equilibrium at a modeled CIP concentration and the control was conducted with a one-sided t-test to determine whether divergence occurred. This step went on until the TCEP could be determined. In order to account for the variability of the parameters, the simplified plankton ecological model was run in a Monte-Carlo setting. The detailed procedure was provided in the supporting document.

### Data Availability

All data generated or analysed during this study are included in this published article (and its Supplementary Information files).

## Results and Discussion

### Impacts of ecological interactions on the toxic effect of CIP

**Figure 1:**
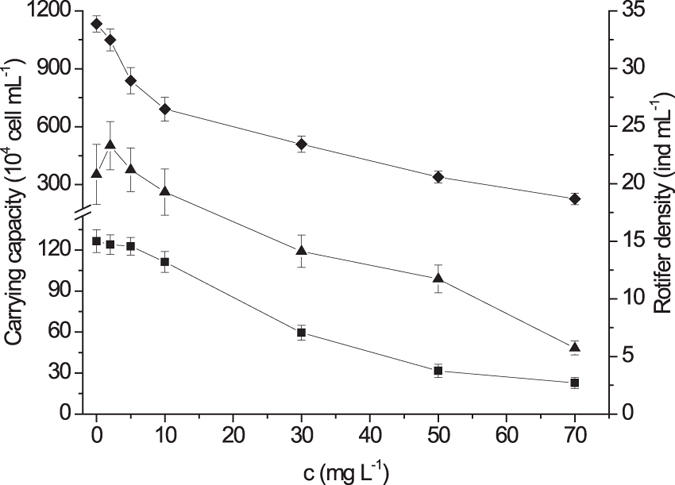
Changes of carrying capacity and rotifer density with Ciprofloxacin hydrochloride concentration in single-species toxicity tests (the dots displaying were mean values and the error bars stood for standard deviations; ■: *P. subcordiformis*; ◆: *I. galbana*; ▲: *B. plicatilis*).

**Figure 2:**
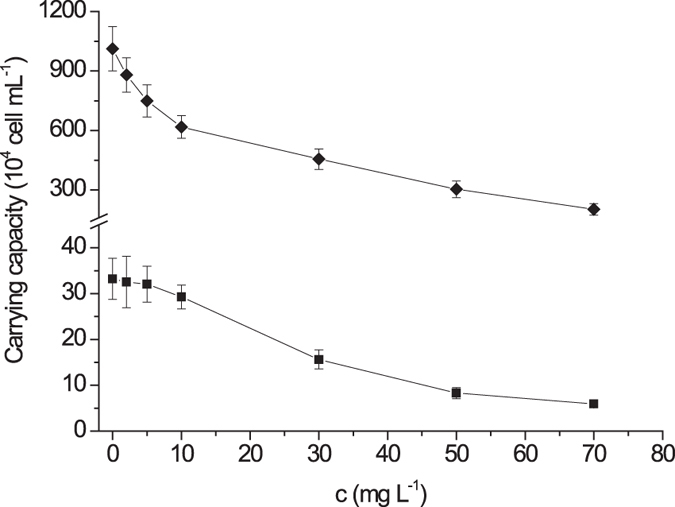
Changes of carrying capacity with Ciprofloxacin hydrochloride concentration in algae bi-species toxicity tests (the dots displaying were mean values and the error bars stood for standard deviations; ■: *P. subcordiformis*; ◆: *I. galbana*).

**Figure 3:**
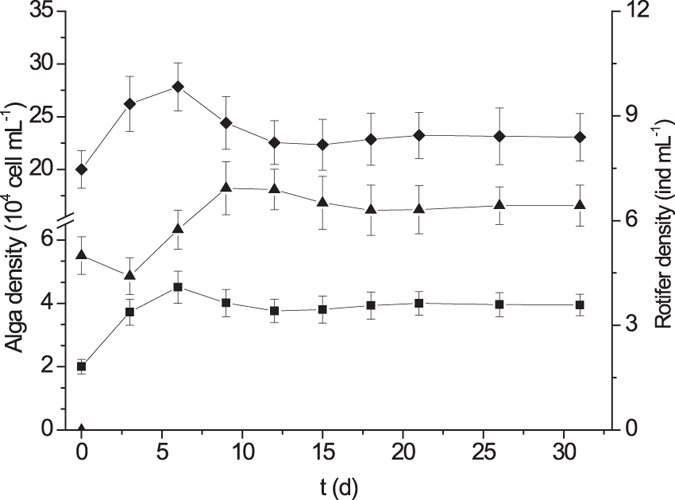
Plankton densities over time inthe unexposed customized plankton community toxicity test (the dots displaying were mean values and the error bars stood for standard deviations; ■:*P. subcordiformis*; ◆: *I. galbana*; ▲: *B. plicatilis*).

The calculated carrying capacity in absence of CIP, was up to 1.27 × 10^6^ cells mL^−1^ in alga single-species test (Fig. 1) and 0.33 × 10^6^ cells mL^−1^ in algae bi-species test for *P. subcordiformis* (Fig. 2), whereas 11.33 × 10^6^ cells mL^−1^ and 10.12 × 10^6^ cells mL^−1^ where the carrying capacity for *I. galbana*, respectively (Figs 1 and 2). This manifested that interspecific competition has resulted in a reduction of 74% in *P. subcordiformis* density and of 11% in *I. galbana* density, indicating the *I. galbana* prevailed over *P. subcordiformis* in their competition. The equilibrium biomasses of three species in the customized plankton community test, was supposed to be approached after 21 days, as densities for each species inclined to stability and did not change significantly over next 5 days (Fig. 3). The equilibrium density of *P. subcordiformis* and *I. galbana* without exposure to CIP in the customized plankton community, were only 0.04 × 10^6^ cells mL^−1^ and 0.23 × 10^6^ cells mL^−1^, respectively. This demonstrated that grazing of *B. plicatilis*has cut down more than 30 times the final algae’s population density, compared with that in algae single species test. In fact, *I. galbana* is found widely in coastal waters of the China Sea^25, 26^. *P. subcordiformis* and *B. plicatilis* occurs worldwide, are extensively found in coastal waters^27–32^. These tested organisms in this study have ever been collected in the same place^33^. *P. subcordiformis* and *I. galbana* are found also in interspecific competition as dominant species in mariculture regions^34^. In this case, it would be interesting and possible to estimate the effect of the antibiotic on the planktonic structure tested in the study.

**Figure 4:**
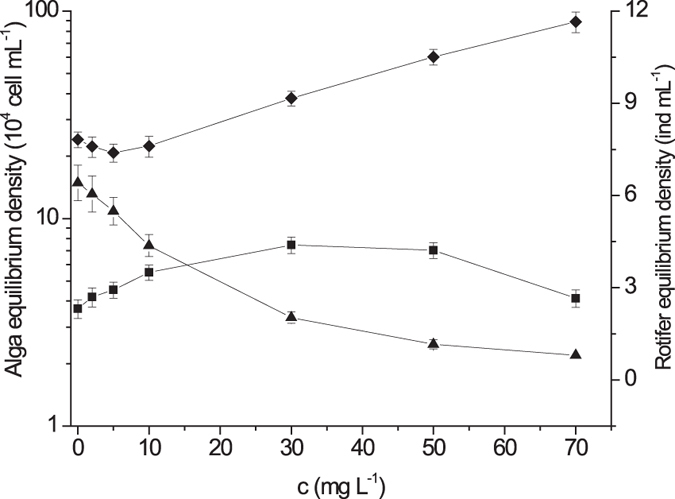
Changes of plankton equilibrium density with Ciprofloxacin hydrochloride concentration in the customized plankton community toxicity test (the dots displaying were mean values and the error bars stood for standard deviations; ■: *P. subcordiformis*; ◆: *I. galbana*; ▲: *B. plicatilis*).

The calculated carrying capacity showed a clear decrement in algae single-species toxicity tests when CIP concentration was higher than 10.0 mg L^−1^ for *P. subcordiformis*, whereas 2.0 mg L^−1^ for *I. galbana* (Fig. 1), demonstrating the ecotoxicological effect of antibiotics on the algal biomasses. And the same patterns of the carrying capacities have been seen in algae bi-species toxicity tests (Fig. 2). The *B. plicatilis* density varied slightly at a concentration lower than 5.0 mg L^−1^ and decreased from concentration higher than 10.0 mg L^−1^ in single-species toxicity test (Fig. 1). A different pattern was found in the customized plankton community toxicity test, where the *B. plicatilis* density decreased from concentration higher than 2.0 mg L^−1^ (Fig. 4).

It is more interesting that the equilibrium densities of *P. subcordiformis* in the customized community, increasing with the CIP concentrations and reaching a peak at 30.0 mg L^−1^, showed a completely different pattern of *I. galbana*, decreasing with the CIP concentrations and falling to a trough at 5.0 mg L^−1^ (Fig. 4). This phenomenon was involved in the well-studied indirect effect, specifically in trophic cascades and interspecific competition in this study, which often occurred jointly and have already been documented in many studies^35–40^. They can explain that the changes in population densities with increasing contaminations in single-species tests are not necessarily same with their densities with in a community due to ecological interactions. With the increasing CIP concentration, a decline of intrinsic growth rate resulted in the decrease in density of *I. galbana*. However, a reduced grazing effect of *B. plicatilis*, which resulted from the reduced rotifers densities with CIP, benefitted the increase of *I. galbana*. Thus a combination of the adverse factor with the favorable one led to a decrease at first and then an increase in density of *I. galbana*, so that an inflexion occurred at 5.0 mg L^−1^. As to *P. subcordiformis*, the reduced grazing effect of rotifers also benefitted its increase in density, and the decline of intrinsic growth rate with CIP brought about a decrease in density in the same way. Nevertheless, as *P. subcordiformis* was less sensitive to the effects of CIP compared to *I. galbana* (Fig. 1), a combination of the two factors led to an increase in density of *P. subcordiformis* at first. In addition, although the *I. galbana* was more competitive than the *P. subcordiformis* (Fig. 2), the interspecific competition effect on *P. subcordiformis* was weak at low CIP concentrations as density of *I. galbana* was also low. The interspecific competition became stronger with the increasing density of *I. galbana* when CIP concentration was higher than 5.0 mg L^−1^, therefore, the enhanced interspecific competition together with the reduced intrinsic growth rate slowed down the increase in density of *P. subcordiformis*, and led to an occurrence of inflexion at a CIP concentration of 30.0 mg L^−1^. The combinations of direct and indirect effects were thus discovered within a customized community in this study.

It was found to be difficult to distinguish the direct effects from the indirect one in such species assemblages^41, 42^. In this study, when concentration of CIP increased from 0 to 10.0 mg L^−1^, alga densityin single-species toxicity test decreased from 1.27 × 10^6^ to 1.11 × 10^6^ cells mL^−1^ for *P. subcordiformis* and 11.33 × 10^6^ to 6.91 × 10^6^ cells mL^−1^ for *I. galbana*, respectively, resulting in a 12% and a 40% reduction in population density (Fig. 1). However, when CIP-related exposure was coupled with interspecific competition, alga density decreased to 0.29 × 10^6^ cells mL^−1^ and 6.17 × 10^6^ cells mL^−1^ in algae bi-species toxicity tests, respectively, then a 77% and a 45% reduction was found (Fig. 2). By comparison, the impact of interspecific competition on algae was distinguished from direct toxic effect. This manifested that competition could have a great impact on toxic effect for *P. subcordiformis*. This scenario also demonstrated the impact of interspecific competition on toxic effect of CIP was different in taxonomy, which may result in changes of community structure.

At the same concentration of 10.0 mg L^−1^ in the customized plankton community toxicity tests, the CIP-related exposures, coupling with grazing and competition, had cut down on the population densities to 0.044 × 10^6^ cells mL^−1^ for *P. subcordiformis* and to 0.22 × 10^6^ cells mL^−1^ for *I. galbana*, causing 96% and 98% reduction, respectively, when compared to that in single-species toxicity tests (Fig. 4). It manifested that the impact of the grazing pressure on CIP-related toxic effect contributed to 19% of decrease in population density of *P. subcordiformis*, and 53% of that of *I. galbana*. And therefore, the impact of trophic cascades was also distinguished from interspecific competition and direct toxic effect. By comparison, it was identified that the impact of the grazing pressure on CIP-related toxic effect on *I. galbana*was dominant, while the impact of interspecific competition on CIP-related toxic effect on *P. subcordiformis* prevailed. Furthermore, the changes of algae densities in the bi-species and community test also manifested that competition and grazing effect on algae density was stronger than toxic effects of CIP at community levels.

Therefore, indirect effects mediated through ecological relations were very pronounced, and these demonstrated that contaminations toxicity coupling with indirect effects would have a remarkable impact on ecosystem function and structure, especially in a low-biodiversity ecosystem. The possible explanation is that species sensitivity cannot be assume to be the same in single species test as in complex test and functional redundancy lacks in the simple ones. In addition, it should be emphasized that this effect is very well described in evolution and similar impact of interspecific interaction are observed on macroalga, mussels, barnacles, crab, amphipods, bacteria, bacterial pathogens in water and sediment^35, 37, 41, 43, 44^, manifesting the wide existence of indirect effects in ecosystems and impossibility to ignore the impact of indirect effects on direct one.

### Impacts of indirect effect on NOEC

One of the targets for the protection of ecosystem is to prevent the obvious variation in every species biomass. The maximum concentration which does not affect the biomass amount of the test species is often necessary to be determined. The No Observed Effect Concentration (NOEC) is a statistic used as an estimate of this concentration. According to the treatments in single-species toxicity test, a NOEC of 5.0 mg CIP L^−1^ was determined for *P. subcordiformis*, 0 mg L^−1^ for *I. galbana* and 10 mg L^−1^ for *B. plicatilis*, respectively, using a one-sided Dunnett test (α = 5%). Current protocols for ecotoxicological effect test and data analysis providing no-effect concentrations or benchmark values of pollutants for environment protection administrator potentially relied on the extrapolation of single-species toxic effect to community-level effect in an assumption of species sensitivity distribution. And the 5% of percentile in species sensitivity distribution was usually defined as the community-NOEC. In this way, an extrapolated community-NOEC was calculated to be 0.5 mg L^−1^.

By the definition of NOEC, a highest test concentration at which the observed parameter does not deviate significantly from the observation in the control^45^, the community-NOEC should be the highest test concentration to which exposure of the customized community will not affect significantly any of the populations. Therefore, by comparison of the treatments in the customized community toxicity test with the control, a community-NOEC of 2.0 mg L^−1^ was determined. This community-NOEC was clearly different from the above extrapolated one, as the former embodied the indirect effects in the customized community and was a result of interspecies interactions, which have partly buffered for the direct effects of CIP^9, 42^. The decreased density of *B. plicatilis* after exposure to a CIP concentration of 2.0 mg L^−1^ led to a less grazing effect on *I. galbana*. A less grazing effect of *B. plicatilis*may likely have counteracted the adverse effect of the CIP-related exposure on *I. galbana* at this concentration. This resulted in an increase in NOEC for *I. galbana*. The favorable change of NOEC indicated that the assessment of ecological risk and derivation of water quality benchmark for antibiotics should integrated ecological interactions by developing more reliable and transparent method.

### TCEP of CIP and its ecological significance

In this study, a simplified plankton ecological model was used to describe the customized community and to calculate the threshold concentration (TCEP) of CIP. The TCPE of the CIP was found to be 1.26 mg L^−1^ with a 95% bootstrapping confidence intervalfrom 1.18 to 1.31 mg L^−1^. It is obvious that the TCPE results differed significantly from the community-NOEC (2.0 mg L^−1^) and the extrapolated one from the single-species toxicity test (0.5 mg L^−1^).

It is well known that phytoplankton is the main primary producer in the marine food chain. Zooplankton is a key link in the marine food chainand plays an important role in energy flux of marine ecosystems. The interspecific competition among phytoplankton and the grazing of zooplankton on phytoplankton are the most representative ecological interactions of marine organisms and constitute the basis for a marine community^46, 47^. Although the plankton community in this study was very simplified, it included the essential constituents and showed the principal ecological reactions in ocean ecosystems. The plankton ecological model constructed in this study has grasped mathematically the key ecological interactions in a realistic marine ecosystem. Therefore, the TCEP of CIP calculated with this model is more relevant ecologically than the extrapolated community-NOEC from the single-species toxicity test on the basis of species sensitivity distributions, and approaches to the real threshold concentration to a certain extent. The TCEP is more remarkable in ecological significance and more reliable in assessment of ecological risk. It is true that the TCEP is still not a real threshold concentration in the natural marine ecosystem, however, TCEP calculation in this study moved forward a small step towards a real threshold concentration at least.

## Conclusion

The toxic effects of CIP on plankton assemblages were tested in this study. The combinations of direct and indirect effects were discovered within a customized community. The results show that indirect effects could have a great influence on toxic direct effect on planktons. The impacts of interspecific competition and trophic cascade could be separated from toxic direct effect by comparison of toxic effects in these tests. And the impact of the grazing pressure on CIP-related toxic effect on *I. galbana* was dominant, while the impact of interspecific competition on CIP-related toxic effect on *P. subcordiformis* prevailed. Furthermore, the indirect effect on algal density was stronger than toxic effects of CIP at this study.

A customized community-based threshold concentration calculated for CIP was different from the extrapolated community-NOEC on the basis of species sensitivity distributions. The beneficial effect was a result of interspecies interactions that buffered for more severe direct effects of toxicants. This demonstrated the indirect effects coupling with antibiotics would have a remarkable impact on ecosystem function and structure. It is impossible to ignore the impact of indirect effects on direct one, especially in a low-biodiversity ecosystem. This effect is very well described in evolution, being an expected response to the results of this research.

## Electronic supplementary material

Supplementary Info File
