# Supplementary material for: Interspecies Interactions Reverse the Hazard of Antibiotics Exposure: A Plankton Community Study on Responses to Ciprofloxacin hydrochloride
Source: Sci Rep. 2017 May 24;7:2373. doi: 10.1038/s41598-017-02593-4 (PMC5443794; doi:10.1038/s41598-017-02593-4)
Supplement: Supplementary file 1 — Supplementary Info File [file 41598_2017_2593_MOESM1_ESM.pdf]

Interspecies Interactions Reverse the Hazard of Antibiotics Exposure: A Plankton Community Study on Responses to Ciprofloxacin hydrochloride

Changyou Wang[1, 5*], Ziyang Wang[2], Yong Zhang[3], Rongguo Su[4]

1. School of Marine Sciences, Nanjing University of Information Science and Technology, Nanjing, 210044, China.

2. Qingdao No.19 Middle School of Shandong Province, Qingdao, 266021, China

3. Institute of Marine Science and Technology, Shandong University, Ji-nan 250100, China

4. College of Chemistry and Chemical Engineering, Ocean University of China, Qingdao, 266100, China

5. Jiangsu Research Center for Ocean Survey Technology, Nanjing University of Information Science and Technology, Nanjing, 210044, China

*Correspondence to Changyou Wang [chywang@nuist.edu.cn]

Supporting document:

In this model, P_1 represents the *P. subcordiformis*, P_2 represents the *I. galbana*, and Z represents the rotifer *B. plicatilis*. In addition, r_i (i=1, 2) denotes the instantaneous growth rate under the experimental conditions, 1 represents the *P. subcordiformis*, 2 represents the *I. galbana*, and r_3 represents the instantaneous death rate of the rotifer *B. plicatilis*. Furthermore, $a_{11} = \frac{r_1}{K_1}$,

$$a_{12} = \frac{\alpha \cdot r_1}{K_1},\ a_{13} = F_1,\ a_{21} = \frac{\beta \cdot r_2}{K_2},\ a_{22} = \frac{r_2}{K_2},\ a_{23} = F_2,\ a_{31} = F_1 \cdot h_1 \text{ and } a_{32} = F_2 \cdot h_2 .$$

K_i (i=1, 2) denotes the carrying capacity of the algae, α is the interspecific competition parameter of *I. galbana* with *P. subcordiformis*, β is the interspecific competition parameter of *P. subcordiformis* with *I. galbana,* and F_i (i=1, 2) is the filtration rate from the zooplankton to the algae (i).

In order to account for the variability of the parameters obtained in the single species toxicity test, the simplified plankton ecosystem model was run in a Monte-Carlo setting. The statistical distribution characteristics describing this variability are shown in Tables 1 and 2. The numbers represent the means of the normal distributions, and the numbers in brackets represent the corresponding standard deviations. First, 1000 parameter data sets used in the simplified plankton ecosystem were obtained with Latin hypercube sampling. Usually, the standard deviations of all parameters stabilized after 100 samplings. Next, five data sets were randomly selected for replacement from the above 1000 parameter data sets. The value of concentration (c_1) was calculated by averaging 0 (no effect concentration) and c_0 (a certain toxic effect concentration). Then, five simulations were run using the five randomly selected data sets at c_1. The results of every population biomass were compared to the corresponding reference experimental data (five control replications), and their significance values were determined using the hypothesis test approach based on Dunnett's test. c_2 was calculated by averaging c_1 and c_0 when c_1 was determined to be a no effect concentration, or by averaging 0 and c_1 when c_1 was determined to be an effect concentration. Subsequently, five simulations were conducted using the five randomly selected data sets at c_2 again. The significance values between the simulations and reference experimental data were determined again, and c_3 was calculated. These steps were repeated until c_m was determined to be a no effect concentration and the difference between c_{m-1} and c_m was small enough to satisfy the required precision.

The confidence intervals (CI95) of c_m were estimated using a bootstrap technique. By applying the same methodology in the previous paragraph, another five data sets were randomly selected for replacement from 1000 parameter data sets, yielding c_m. These procedure were conducted n times, yielding n c_m values. The average value of c_m was calculated to be the threshold concentration of the simplified plankton ecosystem (TCEP); its confidence interval was estimated using the resulting frequency distributions of the pseudo-values (the 2.5th and 97.5th percentile values).

Table 1 Simplified plankton ecosystem parameters

Parameter symbol	Description	Average (standard deviation)
r01	growth rate of *P. subcordiformis* (d^{-1})	0.4 (0.01)
r02	growth rate of *I. galbana*(d^{-1})	0.31 (0.02)
K01	carrying capacity of *P. subcordiformis* (10^6cells mL)	126.5(12.6)
K02	carrying capacity of *I. galbana* (10^4cells mL)	1132.8 (95.1)
α	interspecific competition coefficient of *P. subcordiformis*	0.12(0.02)
β	interspecific competition coefficient of *I. galbana*	7.3 (1.1)
r03	mortality rate of *Brachionusplicatilis* (d^{-1})	0.25 (0.05)
Fa01	filtering rate coefficient for *P. subcordiformis*($ml^2 cell^{-1} ind^{-1} d^{-1}$)	0.016(0.003)
Fa02	filtering rate coefficient for *I. galbana*($ml^2 cell^{-1} ind^{-1} d^{-1}$)	0.002 (0.0003)
W1	dry weight of *P. subcordiformis* (pg $cell^{-1}$)	0.53 (0.04)
W2	dry weight of *I. galbana* (pg $cell^{-1}$)	1.37 (0.15)
W3	dry weight of *B. plicatilis* (μg ind^{-1})	0.28 (0.03)
h1	transfer efficiency from *P. subcordiformis*biomass to *B. plicatilis*biomass (%)	20 (5)
h2	transfer efficiency from *I. galbana*biomass to *B. plicatilis* biomass (%)	20 (5)

Table 2 Toxic effect sub-model characteristics of the customized ecosystem models

Toxic effects	Equation in sub-models	Endpoints	Parameters of sub-models		
			a	b	x0 (mg L^{-1})
Growth effect	$\frac{y}{y_0} = e^{(-a(x^b - x_0^b))}$	K1,r1	0.092 (0.01)	0.75 (0.007)	7.22 (0.9)
		K2,r2	0.27 (0.1)	0.44 (0.1)	1.02 (0.6)
mortality effect	$\frac{1-y}{1-y_0} = e^{(-a(x^b - x_0^b))}$	r3	0.011 (0.005)	1.11 (0.45)	5.0 (0.22)
sublethal toxicant effect	$\frac{y}{y_0} = e^{(-a(x^b - x_0^b))}$	Fa1, Fa2	0.52 (0.05)	0.61(0.08)	0.89 (0.2)
